# A Clinical Lecture on Gastric and Duodenal Ulcer

**Published:** 1915-12

**Authors:** J. L. Campbell

**Affiliations:** Professor of Surgical Anatomy and Clinical Surgery, Atlanta Medical College (Medical Department of Emory University); Visiting Surgeon, Wesley Memorial Hospital, Etc.


### A CLINICAL LECTURE ON GASTRIC AND DUODENAL ULCER.

J. L. Campbell, M. D., F. A. C. S.

Professor of Surgical Anatomy and Clinical Surgery, Atlanta Medical College (Medical Department of Emory University); Visiting Surgeon, Wesley Memorial Hospital, Etc.

Mayo has stated that at least 90 per cent. of all cases of indigestion are due to diseases located in other organs, or to functional disturbances of the stomach. He enumerates as such: cardiac, renal, pulmonary, arterial and many other general diseases, and such functional conditions as neurosis, gastro-enteroptosis, atonic dilatation of the stomach, and lastly, surgical conditions of the gastro-intestinal canal outside the stomach. This would lead us to believe that ten per cent. of all cases of chronic indigestion are due to either gastritis, ulcer, or cancer. Such has not, however, been my observation. I am strongly inclined to put the percentage of true stomach entities much lower, as we see many cases of indigestion relieved by the removal of a chronic appendix or gall stones, or by the drainage of a chronic gall bladder, the breaking up of pelvic adhesions, the repairing of tears in the floor of the female pelvis and the correction of mal-positions of the uterus, etc.

Gastric and duodenal ulcers are far more frequent in some sections of the United States than in the South, and much more common in England and Europe than in America.

A gastric ulcer, like an ulcer in other parts of the body, is "The loss of substance due to molecular death of a superficial structure." (Dacosta) Using this definition as a basis we can readily see how a gastric ulcer may vary in size from a microscopic erosion to several inches in diameter. It may involve only the epithelium, or all the coats of the stomach.

Mayo Robson accepts the division of gastric ulcers into two classes—erosions and simple or true ulcers. Erosions are usually acute. They may involve only the epithelium, being mere abrasions from which the blood oozes more or less freely, or they may destroy the superficial layers and expose the larger vessels in the muscularis mucosa, giving rise to serious or fatal hemorrhage. Erosions may occupy any part of the stomach cavity, and they occur with about equal frequency in both sexes. If there is any difference young women of the servant class are more susceptible to them. As a rule, they yield readily to medical treatment, but may cause fatel hemorrhage within a few hours.

Simple or true gastric ulcers may be divided into acute, non-indurated, and chronic indurated ulvers. The acute non-indurated ulcer, most frequently seen in young women of the chlorotic type, runs a rapid course, either yielding to medical treatment in the majority of cases or causing serious hemorrhage, perforation and death. The chronic indurated type is the kind most often seen by the surgeon.. Classing those situated in the first portion of the duodenum with those in the stomach proper, they will be found much more frequently in men than in women.

The etiology is not definitely settled. Among the predisposing causes, age, sex and occupation play a varying role. Women up to their third decade seem to be more susceptible to acute ulcer than men. After 35 both sexes are more liable to chronic than acute ulcer, and statistics by English authorities place gastric ulcer more frequent in women. In a series of 500 cases in the London Hospital there were 402 in women and 98 in men. In 2,031 cases collected by Fenwick, 1,227 were in women. Seymore Taylor states that he found 72 per cent. in men. The Mayos found 745 ulcers in men in a series of 1,000 cases operated on at St. Mary's Hospital. It is admitted by all authorities that duodenal ulcer is more frequent in men. Ulcer does not seem to select any special class, as the rich and poor are about equally affected.

The exciting cause is thought to be oral sepsis. . Several of the patients seen by the writer have had pyorrhoea alveolaris (Rigg's disease). One can readily see how pus, constantly entering the stomach with each mouthful of food, can lodge in some crypt, or in some point with lowered vitality and cause an ulcer. Poorly masticated food, which is the rule when the gums are sore, is also thought to be responsible, as it requires more hydrochloric acid for its indigestion, and hydrochlide acid in excess is constantly present in ulcer. At any rate, when we consider that 80 to 90 per cent. of chronic ulcers are in the pyloric region or along the lesser curvature, it looks reasonable to suppose that some infection is taken up by the lymphatics and deposited in the glands at these points.

The pathological process is much the same as that in the intestinal lymph nodes during typhoid fever, that is, first, swelling of the infected gland, which is surrounded by leucocytes and other products of inflammation; second, the slough; third the gradual ulceration and digestion of the deeper structures; and, fourth, an attempt to heal, so that the base and edges become indurated. Owing to the presence of hydrochloric acid in the stomach, however, healing is rarely complete.

Chronic ulcers are solitary in 78% of cases while acute ulcers are multiple in about 60%. Ulcers vary greatly in size, averaging, according to Mayo, from the size of a split pea to a dime, but they may attain to several inches in diameter. In one case seen by myself, where hemorrhage had been excessive, the ulcer was only about one-fourth inch in diameter. Mayo states that about 33% of chronic ulcers are gastric; 64% duodenal and 3% are both. In about 6% of cases there are be an erosion on the opposite wall of the stomach. In 1,015 cases of acute and chronic gastric ulcers reported by Fenwick 15.6% were at the pylorus, 36% along the leser curvature; 25% on the posterior wall, 7.9% at the cardia; 4.14%

along the greater curvature, 8% on the anterior wall and 3.3% in the fundus.

The most prominent symptoms presented by a patient suffering with gastric ulcer are as follows:

(1) Pain.
(2) Vomiting.
(3) Hemorrhage, melena or hematemensis.
(4) Indigestion.
(5) Regurgitation, principlaly at night, of sour material.
(6) Hypersecretion and hyperacidity.
(7) Impairment of the general health.

I have tried to enumerate these symptoms in order of their importance from a diagnostic standpoint. The pain will generally be described as gripping, boring and burning in character. It is most intense in the mid epigastric region and referred either to the right, if the ulcer is near the pylorus, or to the left if in the body or near the fundus of the stomach. During an attack, which lasts from several days to a month or more, the pain is most intense about 30 minutes after the intake of food, when the ulcer is acute or proximal to Mayo's line (a line let fall perpendicularly from the cardia). If the ulcer is at the pylorus or in the duodenum the pain is most intense about two to four hours after food is taken, or when the stomach is empty. In this case it is relieved by a glass of milk or an alkaline drink. Patients with duodenal ulcer soon learn that relief may be obtained from the pain which is experienced about two a. m. by drinking a glass of milk and eating a cracker, and will provide these each night. Although pain is perhaps the most constant symptom, many ulcers are found in patients who have never suffered.

Vomiting is also a most prominent and perhaps the next most important symptom. Pain in the acute ulcer and ulcers situated proximal to Mayo's line is relieved by vomiting the stomach contents, yet on the other hand, vomiting may become a serious complication. It may greatly endanger the general health and even death may result. When the ulcer is acute, or during an acute exacerbation of a chronic ulcer, there may be blood in the vomitus, but it is generally the recently taken food, together with a quantity of sour material. If there is pyloric obstruction the food taken for supper may be vomited the following morning—retention vomiting. Patients with chronic ulcer are frequently awaked about two or three o'clock in the morning with a mouth full of sour, bitter fluid and may vomit several ounces each night.

Hemorrhage, which was at one time considered the most important symptom, occurs, according to Mayo, in only about 30% of cases, either in the form of hematemesis or melena. This means that the blood is plainly visible in the vomitus or there are dark colored, tarry stools. Occult blood may be found in many more cases. However, it is easy to find occult blood, as it may be present from the gums or throat or from some abraison in the rectum. It is not safe to expect more than 30% to 40% of cases to give a distinct history of hemorrhage. The blood vomited in ulcer is quite distinct from the dirty coffee-ground material vomited in cancer and one will have little difficulty in distingushing the two conditions.

Hypersecretion and hyperacidity in duodenal ulcer are almost constant symptoms and, like the presence of food in acute gastric ulcer, give great distress when the stomach is empty, as the pylorus is relaxed and the stomach secretion is poured out on the sensitive ulver area in the duodenum. When food enters the stomach two things happen: First, the acid secretion is diluted and, second, the presence of food automatically closes the pylorus for a time, consequently the pain is relieved.

The pressure of gas in the stomach is another constant and annoying symptom causing great distress by the distention. When eructated with a quantity of sour material this frequently causes soreness of the teeth.

So many patients complain of indigestion, that we are in the habit of passing it over lightly but when it has existed for a number of years, and has been "cured" many times, only to recur with more severity, other symptoms should be diligently sought out, especially when there is marked impairment of the general health.

The differential diagnosis is not always an easy matter. In 1906 Graham reported from the Mayo clinic that out of 141 cases of duodenal ulcer, in which the histories were carefully tabulated, that only 67.4% had been correctly diagnosed, 10% were thought to be gall stones, 14.3% either gall stones or ulcer, and 8.3% any one of several conditions. The pain in gall stones, as compared with ulcer, is severe and lancinating. It radiates along the right costal arch and to the right scapular region, is not affected by food, and is seldom relieved by vomiting. There is rarely any blood in the vomitus, except such as may come from the throat or nose.

Appendicitis may be mistaken for gastric ulcer, as it is one of the chief causes of indigestion, and the pain is frequently referred to the epigastric region, but if careful attention is given to the history and points of abdominal tenderness, little difficulty need be experienced.

In renal colic, which is generally accompanied by vomiting, the pain is referred to the urethra and down the thigh; blood will appear in the urine in many cases, while in almost all cases there are pus and crystals of various kinds.

Atonic dilitation and some forms of neurosis may be mistaken for ulcer as there is frequently retention vomiting and sometimes pain.

To recapitulate, the most prominent symptoms on which a diagnosis can safely be based are, first, pain definitely related to food; second, vomiting (a) the stomach contents, which relieves the pain; (b) during the latter part of the night a few mouthfuls of bitter, sour material; third, hematemesis or melena, and, fourth, hypersecretion and hyperacidity.

The X-ray is one of the most useful aids now at hand for completing our diagnosis, as the crater of the ulcer will retain enough of the bismuth to show after the stomach is empty. In the majority of cases analysis of the stomach contents will show an excess of hydrochloric acid.

Mayo Robson enumerates some 23 complications that are likely to occur, but it seems that some of the most important are quite sufficient to remember. Among these are, stenosis, either at the cardia or pyloris. Hour glass contraction and perforations. Adhesions, which are likely to follow, near the pyloris, or on the posterior wall. Acute perintonitis, and localized abscess, due to perforations and, as pointed out by the Mayos, cancer developing in the scar of a healed ulcer.

The prognosis is grave, as will be seen by reports from the London Hospital. In the series of 500 cases collected there and mentioned above, 48 (10%) died from peritonitis, 13 (2½%) died from hemorrhage, 28 (5½%) from other causes. The total death were 89, or approximately 18%, only 32% were discharged as relieved or cured. No less than 42% of the 500 cases had suffered previous attacks. In 116 there had been one attack, in 41 two attacks, in 15 three, and in 39 four or more. In other words, gastric ulcer recurs or relapses in at least two-fifths of all cases.

In another series of 153 patients, six died in the hospital and 147 were discharged as cured. Seventy-two of these have been traced, forty were found still to have definite symptoms; 5 had been operated on for hemorrhage, vomiting, etc., 1 died one year later, and at the postmortem a cancer was found at the ulcer site. Twelve had no symptoms, 5 were probably "not cured" and two others may be cured. Now if sifted down, at least 64%, of cases are not cured by medical treatment.

On the other hand, surgical treatment relieved of all symptoms 90% of the cases and there was a total mortality of only 3% in a series of 300 cases operated on by Mayo Robson.

W. J. Mayo stated that in a series of 1,000 cases the total operative mortality was 2.4%, but since 1906 it had been less than 2% as the technique was better. The prognosis in duodenal and pyloric ulcer is better than in cases where the ulcer is in the body of the stomach, for he had in this class of cases 98% of cures or almost total relief from symptoms, while in the latter only 85% were cured or greatly relieved.

It will be seen by statistics quoted above that medical treatment falls far short of a cure, for even where the best means are employed among the best class of patients, only about 50% can be cured, and these must confine themselves to a selected diet for the remainder of their lives. While this is true of chronic ulcer, erosions and acute ulcers offer a much better prognosis. Rest in bed, restricted diet, soothing alkaline remedies will often give entire relief. It is not wise, except in grave cases, to operate during a period of hemorrhage.

When an operation has been determined upon, there are several methods to be considered. The situation of the ulcer and existing complications must always be kept in mind. In case of pyloric obstructión Finney's pyloroplasty frequently gives good results, but the best results have been obtained by a posterior no loop gastro-jejunostomy with excision or infolding of the ulcer. This is probably best accomplished in a 2 step operation with an interval of two or three weeks, according to the emergency of the case.

It would require entirely too much time to describe in detail the technique of this operation, but a few words will not be out of place. The patient should receive carefule preoperative treatment. In special cases only, such as long standing pyloric stenosis, it is necessary to use lavage and then, for only a few days prior to operation. The mouth should be carefully treated and got in good condition; and sterile food given for several days. Just before the operation a nutrient enema of brandy, liquid peptonoids, or panopepton, with normal saline should be given. These serve as both stimulation and food. The salt solution fills up the vessels and there is some nutrition in the peptonoids.

As above stated, I believe the operation of choice in the majority of ulcer cases is the posterior no ook gastro-jejunostomy. This is easily made and has proven very satisfactory in the large clinics. The abdomen should be opened by an incision long enough to give ample room for free work. The omentum is turned up over the ventral surface of the transverse colon and stomach and the duodenu-jejunal junction located. A rent is made in the transverse meso-colon, selecting a non-vascular area beneath the arteral loops, and the posterior wall of the stomach pushed through sufficiently to allow a section about 3 inches in length to be firmly grasped in the blades of the

Moynihan holding clamps, which should be set so that the incision in the posterior wall of the stomach will be nearly vertical. If there is a mesocolic band extending along the jejunum it should be incised and a section of the gut 3 inches in length, and as near the junction as possible grasped with the clamps. The two clamped sections should be held parallel and a moist pad placed between and behind them to protect the abdominal cavity. As far as possible the withdrawn intestines and omentum should be replaced in the abdomen and a moist pad spread out over the remainder in such a way that the incision in the abdominal wall, and all exposed parts, will be protected before the anastomosis is begun.

The clamp on the stomach and the one on the jejunum are now held firmly side by side. The first row of sutures are laid as close to the blades of the clamp as possible, taking a continuous seromuscular stitch with silk or linen, leaving the ends long enough to continue it around the anterior part of the anastomosis when the clamps are removed. After placing the suture, an incision is made through the peritoneum about one-fourth of an inch on either side, and the two flaps brought together with fine chromic catgut. Lastly, the stomach and intestine are opened and the mucous membrane sutured through and through with chromic catgut, using a lock stitch to control hemorrhage. The Mayos have found that where there was a secondary hemorrhage it occurred along the posterior wall. The last row of sutures is continued around the anterior wall in such a manner that the peritoneal surfaces are brought together. The clamps are removed and the linen sero-muscular suture is continued around to the point of beginning. Great care must be taken to secure the angles firmly, as it is here that leakage is most likely to occur. Murphy has devised an oblong button for this anastomosis and secures most excellent results, but it is claimed that the opening is more liable to contract after the button than after the suture anastomosis. The edges of the opening in the mesocolon should be attached just above the suture line between the stomach and jejunum and the torn edges tucked in. This serves two purposes—if there is leakage it prevents it from entering the lesser cavity, and it also prevents contritacon or an adhesion to the torn margins of the mesocolon. If there is no indication of hemorrhage the abdominal wound may be closed and the patient placed in bed in the Fowler position.

During the first 24 hours as much fluid as can be retained should be given by rectur. I give 12 oz. of about one-half strength normal saline solution with from one-half to one drachm of soda bicarbonate in it every 6 hours until 3 or 4 injections have been given. The second day give liquids in small quantities gradually increasing to thickened broths and solids, by the end of the week a moderate diet may be given. If the patient suffers pain give small doses of morphine and atropine. The bowels may be moved by an enema the second or third day and later with a saline or calomel.

Complications such as nausea, vomiting and obstinate constipation can receive similar treatment for such condition following other operations.

Gastr-ojejunostomy was first performed by Wolflerin, 1891, by bringing a long loop of the jejunum over and attaching it to the anterior wall of the stomach. This is still in use for some conditions. In 1882 Von Hacker did the posterior operation. Many improvements in the technic have been made in the last few years and the operation is now quite free from danger.

We present two patients on whom we have made the diagnosis of gastric ulcer, meaning by this to include both ulcer of the stomach and ulcer of the duodenum. If you will follow their histories closely you can see that they resemble each other very much in the following respects:

(1) The period of time over which the disease had existed.

(2) Attacks of pain definitely related to food.

(3) Vomiting.

(4) Tenderness in the upper abdomen.

(5) Ewalds test meal shows hyperacidity.

(6) The X-ray shows in one, two spots; in the other, one spot, of retained bismuth.

In one, the diagnosis has been confirmed by an operation at Grady Hospital.

Case One.—W. F. A., male, age 44, merchant, married. Family history negative. Was well up to the spring of 1908, when he had an attack of severe pain in the epigastric region, which lasted with varying intensity for several days. He had no further trouble for the next two years, when he had another attack, which was accompanied by severe vomiting. In this attack the pain was so severe that he had to take morphine. These attacks have recurred at varying intervals since, and in the interval between the attacks he has suffered with indigestion, which was thought to be "catarrh of the stomach" and for which he had taken large doses of nitrate of silver

During the early attacks, the pain was greatest about 30 to 40 minutes after eating and was relieved by vomiting. Later the pain was most intense when the stomach was empty and was accompanied by nausea and vomiting of sour material. This pain was relieved when he could retain one or two glasses of milk. He had never vomited blood.

Physical Examination.—Patient rather thin, skin pigmented with the deposits of silver (argyria); liver and spleen negative. No enlarged glands. Has a large, oblique, inquinal hernia on the right side for which we will operate to-day. There is tenderness over upper abdomen with muscular spasm. Tongue heavily coated. Teeth had all been extracted on account of pyorrhoet. Ewalds test meal showed free HCI 35, total acidity 55. Stomach emptied itself, as shown by X-ray, in about four hours, but there was a spot of retained bismuth about one-fourth to three-fourths of an inch above, and to the right of the umbilicus, which practically confirms our clinical diagnosis of gastric ulcer.

Case 2—E. F. H., male, age 55, R. R. flagman, hotel clerk, single. No history of similar trouble in family. Had gonorrhea 25 years ago. No history of syphilis. Has never been jaundiced. Drank freely for several years.

About 7 1-2 years ago had an attack of pain in upper abdomen which lasted several days. Has suffered more or less with indigestion since. Attacks have recurred at varying intervals, and have been accompanied by violent vomiting. Was operated on in New Orleans 5 years ago. Gall bladder region explored but nothing found and no relief experienced. Was in a hospital in Kansas City about 1 year ago, during an attack. Was some better after leaving until about three weeks ago, when an attack of more than ordinary severity came on. There was severe vomiting and at one time a few streaks of blood. Generally constipated, no kidney symptoms.

During all these attacks the pain had been severe and of a gripping nature, frequently requiring morphine for relief. The pain at first, i. e., 6 or 7 years ago, came on within 30 minutes after food. Lately it had been 1 to 2 hours after taking food and lasted until the stomach was empty. Vomiting has always been a prominent symptom but until the last attack there was no blood. Frequently awakes at night with a mouth full of bitter fluid so sour that teeth are "put on edge."

Physical Examination. — Fairly well nourished, nose rather large and streaked with blood vessels. Heart, liver, spleen and lungs negative except aortic second slightly accentuate; tenderness over whole upper abdomen with point of maximum intensity in medium line about 1 inch above umbilicus, scar over gall bladder, no enlarged glands, urine, sp. gr. 1.030, otherwise negative.

Stomach contents: after Ewalds test meal, 6 oz. well digested: Total acidity,, 85; Free HCl. 55; Combined acids, 25; Acid salts, 5; occult blood, negative; lactic acid, negative.

X-ray shows two small spots of retained bismuth just to right of median line and a little above umbilicus.

Operation, Grady Hospital Feb. 26, 1915. Long median incision. Pylorus, gall bladder, abdominal wall and under surface of liver so matted together with adhesions that it was impossible to separate them. Two hard masses were felt and explored with a long needle but found to be only fibrous. A posterior no loop gastro-jejunostomy was done, and abdomen closed. Patient vomited only one time and made an uneventful recovery. The mass was thought to be malignant but patient is quite well and strong and suffers comparatively little and has gained flesh. Is now running a prosperous hotel.

Reference, W. J. Mayo, Boston M&S Journal, April 6, 1911. Annals of Surgery, June 1900; St. Paul Med Journal, Jan., 1911; Annals Surgery, Sep., 1911; Annals Surgery, Jan., 1912.

MacCarty Annals Surgery, May, 1910.

Graham, Jour., Minn. St. Med. Asso. & N W Lancet, Apr. 1, 1910.

Osler's Practice of Medicine.

Mayo Robson, in Keens Surgery.

Dacostas Surgery.

Robt Milne, Murphy's Clinics, Vol. II, p. 223.

Cannon, Amer. J. of Med., Sc., April, 1906

Wyeth's Surgery.

